# Molecular Anatomy of the Class I Ligase Ribozyme for Elucidation of the Activity-Generating Unit

**DOI:** 10.3390/biology12071012

**Published:** 2023-07-17

**Authors:** Miho Kasuga, Hiromi Mutsuro-Aoki, Tadashi Ando, Koji Tamura

**Affiliations:** 1 Department of Biological Science and Technology, Tokyo University of Science, 6-3-1 Niijuku, Katsushika-ku, Tokyo 125-8585, Japan; kasuga.miho34@gmail.com (M.K.); mutsuro@rs.tus.ac.jp (H.M.-A.); 2 Department of Applied Electronics, Tokyo University of Science, 6-3-1 Niijuku, Katsushika-ku, Tokyo 125-8585, Japan; tando@rs.tus.ac.jp; 3 Research Institute for Science and Technology, Tokyo University of Science, 2641 Yamazaki, Noda, Chiba 278-8510, Japan

**Keywords:** class I ligase ribozyme, minimization, RNA, RNA world, origin of life

## Abstract

**Simple Summary:**

The class I ligase is an in-vitro-evolved ribozyme with a high catalytic turnover. In the present study, we considered the conditions under which this ribozyme retains ligation activity by removing the partial structure and by splitting. The ligation activity was maintained even when the structure was split into two molecules of 55 and 39 nucleotides. Our study clarified in several cases the length of the duplexes that is necessary to facilitate activity of the class I ligase ribozyme assembled from multiple fragments.

**Abstract:**

The class I ligase ribozyme consists of 121 nucleotides and shows a high catalytic rate comparable to that found in natural proteinaceous polymerases. In this study, we aimed to identify the smaller active unit of the class I ligase ribozyme comprising ~50 nucleotides, comparable to the estimated length of prebiotically synthesized RNA. Based on the three-dimensional structure of the class I ligase ribozyme, mutants were prepared and their ligation activities were analyzed. Sufficient ligation activity was maintained even when shortening to 94 nucleotides. However, because it would be difficult to approach the target of ~50 nucleotides by removing only the partial structure, the class I ligase ribozyme was then split into two molecules. The ligation activity was maintained even when splitting into two molecules of 55 and 39 nucleotides. Using a system with similar split ribozymes, we analyzed the ligation activity of mutants C30, C47, and A71, which have been previously identified as the positions that contribute to catalytic activity, and discussed the structural basis of the activity of these bases. Our findings suggest the rationale for the class I ligase ribozyme’s assembling from multiple fragments that would be achievable with prebiotic synthesis.

## 1. Introduction

All life phenomena on Earth are understood on a common concept of the “central dogma” [1]—the idea that the genetic information stored in DNA is transcribed into RNA, which is then translated into proteins to transmit the information necessary for life activities in one direction. However, considering the evolution of genetic information transmission on primitive Earth, it is unlikely that the complex systems of DNA, RNA, and proteins of modern life have suddenly emerged. Furthermore, DNA, which is a source of information, is required for protein synthesis; however, proteins are also required for the transcription and translation of DNA. This raised the so-called “chicken-or-egg” problem, that is, whether DNA or protein occurred first in primitive Earth. However, reverse transcriptases have been discovered [2], and it has been proven that RNA plays a role in retaining genetic information, such as DNA. In addition, the discovery of RNA with enzymatic activities [3,4] has led to the proposal of the RNA world hypothesis, which states that RNA is responsible for both the retention of genetic information and enzyme activity on primitive Earth and that RNA alone may have established a self-replicating system [5,6].

For RNAs to be diverse in the RNA world, it is important for small RNAs to ligate and form larger structures. A complete library consisting of one copy each of all possible nucleotides based on monomer incorporations would require more weight than the detour of short RNA fragment ligations. Therefore, the original self-replicators may have utilized simple, template-directed oligonucleotide ligation [7,8]. The discovery of ligase ribozymes [9,10,11,12,13,14,15] makes it likely that ligase functionality could have been acquired even within a limited sequence space. (In the laboratory use of oligonucleotides on the order of 100 µg, the complexity of this pool corresponds to approximately 10^15^ individual sequences [16].) Based on this idea, we focused on the ligation activity of RNA. Several ligation ribozymes have been isolated from a large pool of random sequences [16,17], including the R3C ligase [9], L1 ligase [10], DSL ligase [11], and class I ligase ribozymes [12,13,14,15]. The R3C and L1 ribozymes have already been minimized [18,19,20], and kissing-loop interaction-mediated conformational changes have been shown to acquire activity from small non-active RNAs in the R3C ribozyme [21,22,23,24].

**Figure 1:**
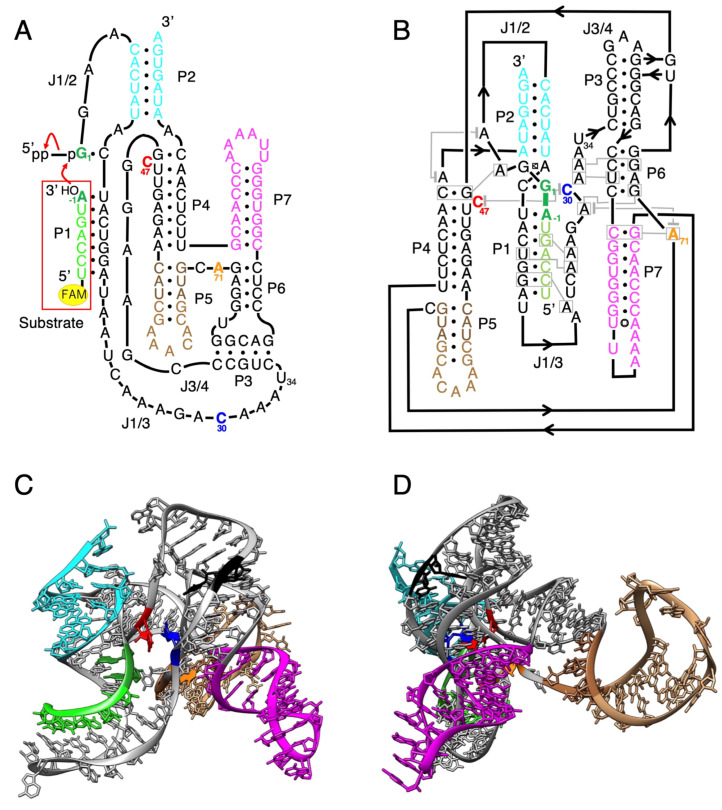
Structure of the class I ligase ribozyme. (**A**) Composition of the class I ligase ribozyme with a fluorescence-labeled RNA substrate. The ribozyme is composed of 121 nucleotides and forms 7 paired regions (P1–P7) by binding the substrate. A 5′-FAM-labeled substrate is indicated by a red rectangle. Red arrows indicate an attack by the substrate 3′-hydroxyl on the ribozyme α-phosphate with a concomitant loss of pyrophosphate. C30, C47, and A71 are suggested as important residues and colored in blue, red, and orange, respectively. The substrate, P2, P5, and P7 are colored in light green, cyan, brown, and magenta, respectively. This figure was modified from Shechner et al. [25]. Although this is a classical secondary-structure representation [33], we use this representation throughout this paper to facilitate the understanding of the substructure of the ribozyme. (**B**) Revised secondary structure based on the crystallization construct after substrate ligation, which was modified from Shechner et al. [25]. The same sequence at the P5 region as in Figure 1A is used in this figure. Indicated is the ligation junction (thick green dash), base triples (boxed residues connected with gray lines), and stacking interactions (residues vertically aligned or connected with gray lines terminating in gray bars). (**C**) Tertiary structure of the class I ligase ribozyme (PDB ID: 3HHN). The ligation junction (G1 and A−1), C30, C47, and A71 are colored in green, blue, red, and orange, respectively. U34 and C35 are colored in black, where the split was performed (also shown in Figure 7 below). The substrate, P2, P5, and P7 are colored in light green, cyan, brown, and magenta, respectively. The P5 region in the crystal structure was replaced with the U1A-binding loop and the sequence is 5′-CAUACCCAUUGCACUCCGGGUAUG-3′. The underlined parts (also shown in light brown) are different from the sequence shown in Figure 1A,B. (**D**) Side view of the tertiary structure of the class I ligase ribozyme, rotated approximately 90° relative to Figure 1C, which highlights the P5 region.

Although these ligase ribozymes basically link multiple fragments of RNA together, the nucleophilic attack by a 3′-hydroxyl on a 5′-α-phosphorus of triphosphates to form a 3′-5′-phosphodiester bond is the same as the replicating process with RNA polymerases. The class I RNA ligase ribozyme (Figure 1) [12,13,14,15] has a catalytic rate among the fastest of known ligase ribozymes, and a model indicates that catalysis with the class I ligase resembles the mechanism of proteinaceous enzymes that replicate RNA [25]. This has been improved by mutation and selection, and the known catalytic RNAs with activities required for general RNA replication are derived from class I ligases. However, although the crystal structure of the class I RNA ligase shows exactly how the α-phosphate of the GTP is positioned with respect to the 3′-hydroxyl of the extended primer, and how Mg^2+^ ions are positioned to catalyze the reaction [25,26], it is not clear what kinds of structural features contribute to effectiveness for the assembling of the active ribozyme. Manfred Eigen’s concept of a “hypercycle” argues that nucleotide lengths must be less than 100 nucleotides for self-replication to occur without an error-correcting mechanism [27]. Moreover, the length of RNA naturally synthesized using the clay mineral montmorillonite has been shown to be approximately 50 nucleotides [28]. In addition, a minimized L1 ligase ribozyme only contained a catalytic core of ~35 nucleotides [20,29], showing that relatively small RNA fragments (even less than 50 nucleotides) could also have participated in a prebiotically plausible RNA ligation.

The concept that a ribozyme can assemble from multiple fragments and retain functionality has been proved for many ribozymes, including the R3C ligase ribozyme [9], the *Azoarcus* group I intron ribozyme [30], the polymerase ribozyme [31], and a self-triphosphorylation ribozyme [32], to name a few. Several of these studies already elaborated on the idea that shorter fragments would be more abundant in a prebiotic environment than longer fragments. However, the class I ligase exhibits a high catalytic rate of up to 360 per minute and it is important to study the minimum structural requirement that is necessary to facilitate such a high activity of the ribozyme.

With these in mind, we aimed to identify the smallest active unit of the class I ligase ribozyme and the rationale for the reaction underlying the structure.

## 2. Materials and Methods

### 2.1. Preparation of Class I Ligase Ribozyme, Its Mutants, and RNA Substrate

Unlabeled deoxyribonucleotides were synthesized with Eurofins Genomics (Tokyo, Japan). An HPLC-purified 5′-terminal 6-carboxyfluorescein (6-FAM)-labeled oligonucleotide (5′-FAM-UCCAGUA-3′) was prepared by Japan Bio Services Co., Ltd. (Saitama, Japan). Each template DNA was prepared from chemically synthesized deoxyribonucleotides carrying the T7 promoter, sequences corresponding to variants of the class I ligase ribozyme, and two synthetic primers using a polymerase chain reaction. RNA transcription was performed at 37 °C for 16 h in a reaction mixture containing 40 mM of Tris-HCl (pH 8.0), 10 mM of dithiothreitol, 2 mM of spermidine, 8 mM of MgCl_2_, 2.5 mM of each NTP, template DNA (0.2 mg/mL), and a pure T7 RNA polymerase (~100 μg/mL) [34]. Although a T7 RNA polymerase extends 3′-termini during run-off transcription, transcripts were carefully purified with denaturing 12% polyacrylamide gel electrophoresis. Because the ligation site is between the 3′-OH of the chemically synthesized, HPLC-purified 5′-FAM-labeled substrate and the 5′-α-phosphorus of triphosphates of the RNA transcript, we judged that the ligation itself was not affected much even if any small amount of the 3′-extended RNA transcript was contaminated. The concentrations of the purified RNA were determined from UV absorbance at a wavelength of 260 nm using an Implen NanoPhotometer (München, Germany).

### 2.2. Analysis of Ligation

**Figure 2:**
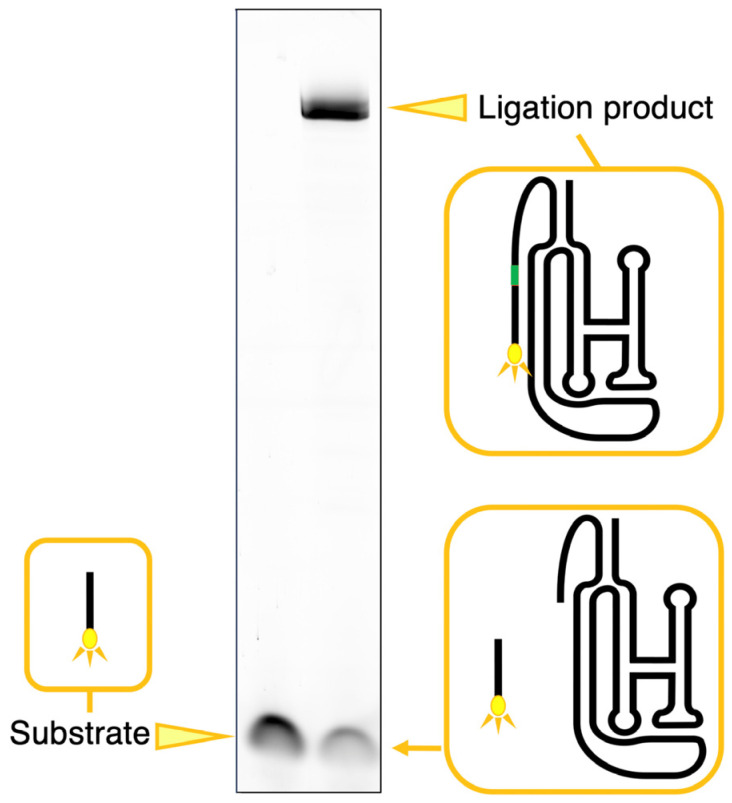
Typical example of the detection of the ligated RNAs and quantitation with the Typhoon imager. Compared with the position of the fluorescence band of the 5′-FAM-labeled substrate, the band of RNA ligated with the substrate appears in the upper part of the denatured polyacrylamide gel in electrophoresis according to the nucleotide length.

A ligation analysis was performed using the method described by Rogers and Joyce, with slight modifications [9]. The class I ligase ribozyme or its variants dissolved in a solution containing 50 mM of Tris-HCl (pH 7.0), 10 mM of MgCl_2_, and 50 mM of KCl were first heated to 37 °C for 5 min and then cooled to 4 °C. The ligation reaction was initiated by adding 3 µL of a 10 µM 5′-FAM-labeled substrate to the solution. The final concentrations of the ribozyme and 5′-FAM-labeled substrate were 1 and 2 µM, respectively. The reaction mixture volume was 15 µL. After incubation at 23 °C for 18 h, the solution was denatured on a 12% polyacrylamide gel for electrophoresis (Figure 2). For the time courses analysis, after incubation at 23 °C for the indicated time, 15 μL aliquots were rapidly frozen, and electrophoresis was performed for all samples together in the same manner. The gel was analyzed on a Typhoon FLA 7000 (GE Healthcare Japan, Tokyo, Japan) by reading fluorescent pigment label samples, and the ligated products were quantified using Image Quant TL software (version 8.2.0.0).

## 3. Results

### 3.1. Ligation Activities of Class I Ribozyme and Deletion Mutants in Regions P7 and P5

**Figure 3:**
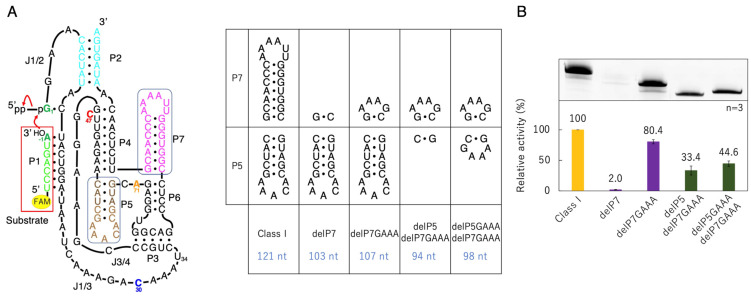
Ligation activities of the class I ribozyme and deletion mutants in regions P7 and P5. (**A**) Secondary structures of the designed P7 and P5 mutants. (**B**) Green bands (λ_ex_ = 473 nm, Y520 filter) associated with 6-FAM-labeled RNAs were detected in denaturing 12% polyacrylamide gel electrophoresis by analyzing on a Typhoon FLA 7000 (top). The ligated products were quantified by using Image Quant TL software. The activities are shown as relative values (%) compared to those in the case of the full-length class I ligase ribozyme (100%). Error bars represent the standard deviation of triplicate experiments (bottom).

From the secondary (Figure 1A,B) and three-dimensional structure (Figure 1C,D) of the class I ligase ribozyme, it was thought that the ligation active site would not be affected, even if the P7 and P5 parts were removed. Therefore, we prepared a mutant in which the P7 moiety was completely removed (delP7, 103 nucleotides); however, the activity was significantly reduced (Figure 3). Then, a P7 deletion mutant with the GAAA tetraloop, which is a type of tetraloop that stabilizes the structure of RNA [35], was prepared (delP7GAAA, 107 nucleotides), and a ligation reaction was performed. The activity of delP7GAAA was maintained at approximately 80% of that of the original class I ligase (Figure 3). Therefore, we used delP7GAAA as the P7 deletion mutant reference.

Next, based on delP7GAAA, we prepared a mutant in which the P5 moiety was completely removed (delP5 delP7GAAA, 94 nucleotides) and a mutant with GAAA of the P5 deletion mutant (delP5GAAA delP7GAAA, 98 nucleotides) (Figure 3). The activity of delP5GAAA delP7GAAA remained higher than that of delP5 delP7GAAA, but the difference was not significant compared to delP7 and delP7GAAA (Figure 3). Therefore, we decided to prepare a mutant by completely removing the P5 region (without the GAAA loop) as the P5 deletion mutant reference.

### 3.2. Ligation Activities of Class I Ribozyme and Deletion Mutants in Regions P2, P4, and J3/4

**Figure 4:**
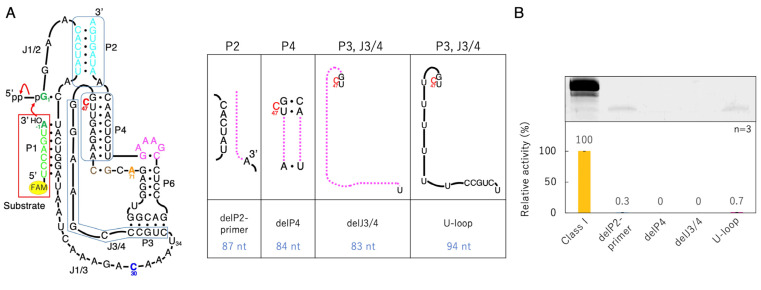
Ligation activities of the class I ribozyme and deletion mutants in regions P2, P4, and J3/4. (**A**) Secondary structures of the designed P2, P4, and J3/4 mutants. The region in magenta dotted lines was deleted from delP5 delP7GAAA shown to the left. (**B**) Green bands (λ_ex_ = 473 nm, Y520 filter) associated with 6-FAM-labeled RNAs were detected in denaturing 12% polyacrylamide gel electrophoresis by analyzing on a Typhoon FLA 7000 (top). The ligated products were quantified by using Image Quant TL software. The activities are shown as relative values (%) compared to those in the case of the full-length class I ligase ribozyme (100%). Error bars represent the standard deviation of triplicate experiments (bottom).

Based on delP5 delP7GAAA, mutants with mutations in the P2, P4, and J3/4 regions were prepared, and ligation reactions were performed (Figure 4). First, seven nucleotides at the 3′-end of the P2 region were deleted (delP2-primer, 87 nucleotides). And because the C47 has been pointed out to be directly involved in the ligation reaction [25], a P4 deletion mutant was prepared by keeping C47 (delP4, 84 nucleotides). For the J3/4 region, a mutant excluding one side of the P3 base pairs and the joint region between the P3 and P4 moiety (delJ3/4, 83 nucleotides), and a mutant in which all J3/4 were replaced with U (U-loop, 94 nucleotides), were prepared (Figure 4). All the mutants in the P2, P4, and J3/4 regions exhibited significantly reduced activity. Slight activity was observed with the delP2-primer and U-loop, but no fluorescent bands were observed with delP4 and delJ3/4 under our experimental conditions (Figure 4). In our experimental conditions using the 5′-FAM-labeled substrate, background levels of our system derived from the invisibly small columns analyzed with Image Quant TL are the same as those derived from the system of the annealing of a 5′-phosphate next to a 3′-hydroxyl, which was conducted by Rohatgi et al. [36]. Therefore, the activity is marked as 0 in Figure 4.

### 3.3. Ligation Activities of Mutants Split into Two Molecules

**Figure 5:**
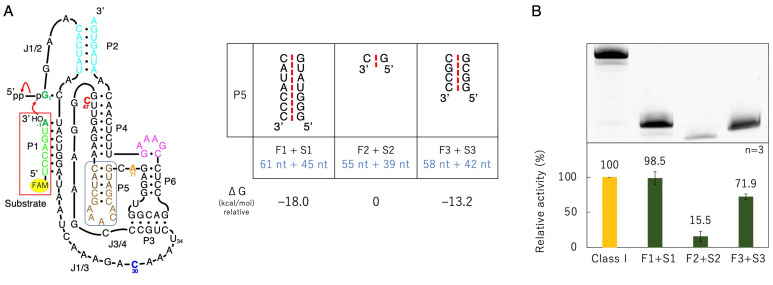
Ligation activities of the class I ribozyme and mutants split into two molecules. (**A**) Secondary structures of the designed split mutants. Splits were conducted on delP7GAAA (shown to the left), becoming two molecules at the P5 region. The red dotted line symbolically represents the boundary between the two split molecules (top right). The free energies for forming a duplex at the split P5 region were estimated. The thermodynamic contributions to helix formation were based on the estimation by Tinoco Jr. and coworkers [37] (bottom right). (**B**) Green bands (λ_ex_ = 473 nm, Y520 filter) associated with 6-FAM-labeled RNAs were detected in denaturing 12% polyacrylamide gel electrophoresis by analyzing on a Typhoon FLA 7000 (top). The ligated products were quantified by using Image Quant TL software. The activities are shown as relative values (%) compared to those in the case of the full-length class I ligase ribozyme (100%). Error bars represent the standard deviation of triplicate experiments (bottom).

**Figure 6:**
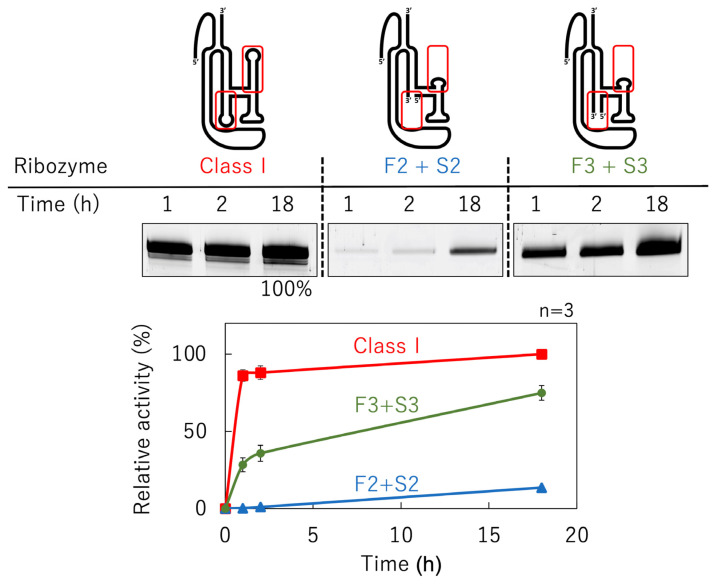
The time courses of the ligation activities of the class I ribozyme and the split mutants. Schematic presentation of the class I ribozyme and mutants split into two molecules (top). The details of the secondary structures are shown in Figure 5. After incubation at 23 °C for the indicated time, 15 μL aliquots were rapidly frozen, and finally all samples were applied together to denaturing 12% polyacrylamide gel electrophoresis. The gel was visualized on a Typhoon FLA 7000 (middle). The ligated products were quantified using Image Quant TL software and plotted on the graph. The activities are shown as relative values (%) compared to those in the case of the full-length class I ligase ribozyme (100%). Error bars represent the standard deviation of triplicate experiments (bottom).

We split delP7GAAA into two molecules in the P5 region (Figure 5). Mutants with seven, one, and four base pairs in the P5 region were composed of First1 + Second1 (F1 + S1, 61 + 45 nucleotides), First2 + Second2 (F2 + S2, 55 + 39 nucleotides), and First3 + Second3 (F3 + S3, 58 + 42 nucleotides), respectively (Figure 5). The reaction time course is shown in Figure 6. The class I ligase ribozyme completed the ligation at nearly 90% in approximately 1 h under the reaction conditions used, whereas F2 + S2 and F3 + S3 were gradually ligated as the reaction time increased (Figure 6). Although reduced activity was observed even in the case of the shortest separate pair, F2 + S2, we decided to use mutants based on F3 + S3 constructs to facilitate the comparison of mutant analyses (Figure 5).

### 3.4. Ligation Activities of Deletion Mutants Based on F3 + S3

**Figure 7:**
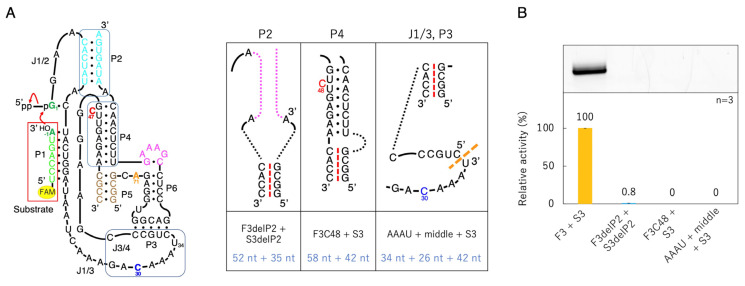
Ligation activities of deletion mutants based on F3 + S3. (**A**) Secondary structures of the designed mutants. The P2 region in magenta dotted lines was deleted from F3 + S3 (shown to the left). The red dotted line symbolically represents the boundary between F3 and S3. The orange dotted line indicates the split position in F3. The corresponding position is also indicated in Figure 1C,D in black. (**B**) Green bands (λ_ex_ = 473 nm, Y520 filter) associated with 6-FAM-labeled RNAs were detected in denaturing 12% polyacrylamide gel electrophoresis by analyzing on a Typhoon FLA 7000 (top). The ligated products were quantified by using Image Quant TL software. The activities are shown as relative values (%) compared to those in the case of the full-length class I ligase ribozyme (100%). Error bars represent the standard deviation of triplicate experiments (bottom).

To minimize F3 + S3, mutants of the P2, P4, and J1/3-P3 regions were generated (Figure 7). In the P2 region, we deleted base pairs (F3delP2 + S3delP2, 52 + 35 nucleotides). In the P4 region, C47 was designed to shift to the 48th position to confirm the importance of C47 (F3C48 + S3, 58 + 42 nucleotides). In the J1/3-P3 region, F3 was split into two parts (AAAU + middle + S3, 34 + 24 + 42 nucleotides) (Figure 7). These three mutants exhibited significantly reduced activity. F3delP2 + S3delP2 showed slight activity, but F3C48 + S3 and AAAU + middle + S3 did not show any fluorescent bands under our experimental conditions (Figure 7).

### 3.5. Ligation Activities of Mutants with Active Site Substitution Based on F3 + S3

**Figure 8:**
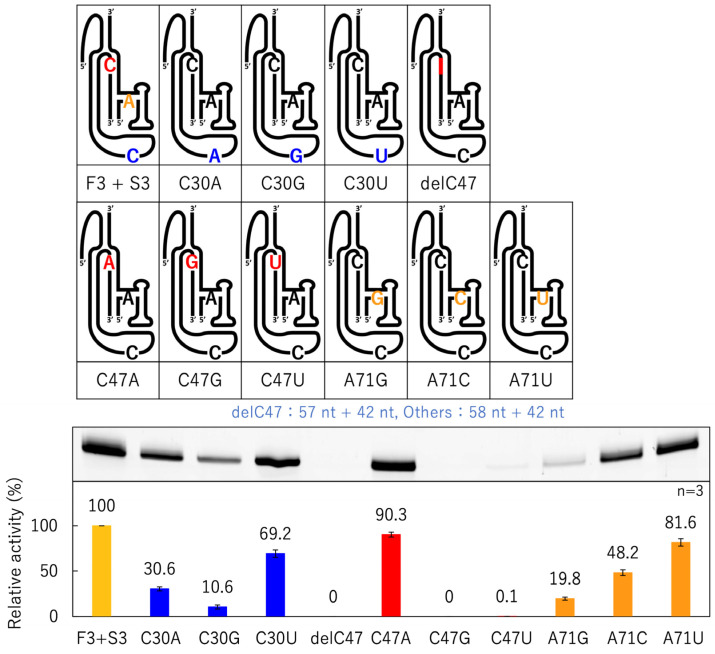
Ligation activities of mutants with active site substitution based on F3 + S3. Schematic presentation of F3 + S3 and its mutants at positions 30, 47, and 71 (top). Green bands (λ_ex_ = 473 nm, Y520 filter) associated with 6-FAM-labeled RNAs were detected in denaturing 12% polyacrylamide gel electrophoresis by analyzing on a Typhoon FLA 7000 (middle). The ligated products were quantified using Image Quant TL software. The activities are shown as relative values (%) compared to those in the case of F3 + S3 (100%). Error bars represent the standard deviation of triplicate experiments (bottom).

To confirm whether the combination of C30, C47, and A71 at the active site was optimal, as inferred from the tertiary structure [25], mutants in which each base was substituted were prepared for F3 + S3 (Figure 8). To confirm the importance of the bulged-out structure of C47, a deletion mutant of C47 was also prepared (Figure 8).

The C30 substitution had a greater effect on ligation activity in the order of G, A, and U, and the A71 substitution had a greater effect on ligation activity in the order of G, C, and U, but the activity was lower than that of F3 + S3, regardless of which base was replaced (Figure 8). When C47 was replaced with U, the ligation ratio was only 0.1%, and when it was removed or replaced with G, the fluorescent band could not be confirmed under our experimental conditions. However, when A was replaced, ligation activity was similar to that of F3 + S3 (Figure 8).

### 3.6. Ligation Activities of Mutants with the 47th Base Fixed to A

**Figure 9:**
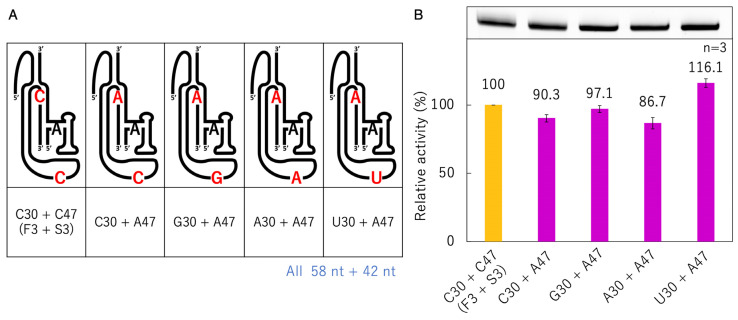
Ligation activities of mutants with the 47th base fixed to A. (**A**) Schematic presentation of F3 + S3 and its mutants at positions 30 with constant A47. (**B**) Green bands (λ_ex_ = 473 nm, Y520 filter) associated with 6-FAM-labeled RNAs were detected in denaturing 12% polyacrylamide gel electrophoresis by analyzing on a Typhoon FLA 7000 (top). The ligated products were quantified by using Image Quant TL software. The activities are shown as relative values (%) compared to those in the case of F3 + S3 (100%). Error bars represent the standard deviation of triplicate experiments (bottom).

Because the activities of the mutants of C47 were almost undetectable, except when C47 was replaced with A, showing an activity close to that of F3 + S3 (Figure 8), further mutants were prepared in which the 47th position was fixed to A and C30 was replaced with other bases (Figure 9). The activity was similar to that of F3 + S3, even when the 30th position was replaced with any other base. In particular, the activity of U30 + A47 slightly exceeded that of F3 + S3 (Figure 9).

## 4. Discussion

The class I ligase is an in-vitro-evolved ribozyme with high-performance capability, analogous to a “Ferrari” [38]. Under optimal reaction conditions, the catalyst exhibited a catalytic rate of up to 360 per minute. However, similar to a Ferrari, it is very forgiving and is fine-tuned to achieve the maximum performance. From an evolutionary point of view, the present study, which considers the conditions under which this ribozyme is split and generates ligation activity, is of great significance, as was the case in our previous series of studies on the R3C ligase ribozyme [19,21,22,24].

The ligation activity of the class I ligase was maintained when the P7 region was replaced with the GAAA tetraloop, whereas the deletion of the P7 region lost its ligation activity (Figure 3). However, neither the deletion nor GAAA capping of the P5 region significantly affected ligation activity (Figure 3). This suggests that stabilization at the edge of the P7 region is important for activity but that the P5 region is not involved in structural stabilization. However, because split mutants in the P5 region showed ligation activities depending on the length of Watson–Crick base pairings (F1 + S1, F2 + F2, and F3 + S3) (Figure 5), tethering through the P5 region was effective for the construction of the structure of the ligase ribozyme. The contributions of 5′-GG-3′/5′-CC-3′, 5′-GU-3′/5′-AC-3′, 5′-UA-3′/5′-UA-3′, 5′-AU-3′/5′-AU-3′, 5′-UG-3′/5′-CA-3′, 5′-GC-3′/5′-GC-3′, and 5′-CG-3′/5′-CG-3′ to ΔG are −5.0, −2.2, −1.8, −1.8, −2.2, −5.0, and −3.2 kcal/mol, respectively [37]. Therefore, F1 + S1 and F3 + S3 would be thermodynamically stabilized using −18.0 and −13.2 kcal/mol, respectively, relative to F2 + S2 (Figure 5A). Thus, the results of the split molecules suggested that simpler synthesized RNA strands may have been linked to have various functions and create diversity. Regarding the other parts, the 3′-end of the P2 region is considered to contribute so that the ribozyme is more reliably folded and forms a three-dimensional structure using the base pairing (Figure 4). The P4 and J3/4 regions are also thought to be necessary for stabilizing the structure near the active ligation site (Figure 4).

**Figure 10:**
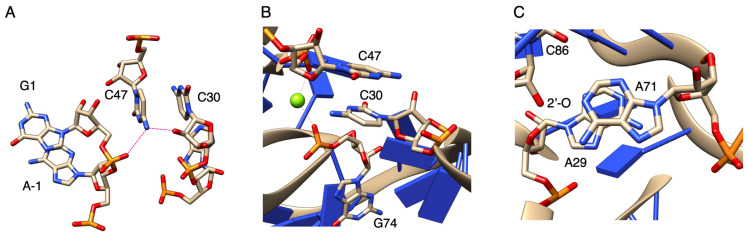
The class I ligase active site (PDB ID: 3HHN). (**A**) View of the active site around C47. The amino group of C47 is thought to be hydrogen bonded with the 2′-oxygen of the ribose of C30 and the α-phosphate oxygen of G1. (**B**) View of the active site around C30. An Mg^2+^ ion, shown as a magenta sphere, coordinates with the phosphate oxygens of C47 and G74. (**C**) View of the area including the stacking interaction between A29 and A71. The 2′-oxygen of the ribose of C86 is also labeled.

When each base was substituted at the active site of F3 + S3, the activity was not retained when C47 was removed or replaced with G or U; however, the activity was close to that of F3 + S3 only when replacing with A (Figure 8). Similar to C and A, the amino group outside the ring can form a proton-donating hydrogen bond, and N3 in the C ring or N1 in the A ring can form a proton-accepting hydrogen bond [39]. On the other hand, in both G and U, the carbonyl oxygen can form a proton-accepting hydrogen bond, and N1 in the G ring or N3 in the U ring can form a proton-donating hydrogen bond [39]. Thus, it is considered that the amino group of C or A at the 47th position is close to the distance at which G1 and substrate A−1 can be ligated by forming a proton-donating hydrogen bond with the 2′-oxygen of the ribose of C30 and the α-phosphate oxygen of G1 (Figure 10A).

In the case of the A47 mutants, the substitution of C30 with other bases caused activities close to those of the original F3 + S3 (Figure 9). This was in contrast to the results for C47 with N30 (N = A, C, G, U), which showed a large decrease in activity with purines at the 30th position (Figure 8). Originally, Mg^2+^ ions coordinated with the phosphate oxygens of C47 and G74; however, the substitution of C30 with purines could affect the coordination of Mg^2+^ (Figure 10B). C30 has a stacking interaction with A47 (Figure 10A), but by converting C30 to purines, the stacking interaction with the 47th base could become stronger. Regardless of the type of base, it would gain the same stability as the effect obtained with Mg^2+^ coordination, even if the coordination is lost (Figure 10B). The stacking interaction between A29 and A71 can also bring G1 closer to substrate A−1 (Figure 10C). Substituting A71 for G71 or C71 may affect the stacking interaction because of the repulsion between the carbonyl oxygen at the 6th position of G71 and the 2′-oxygen of the ribose of C86 and with the repulsion between the amino group at the 4th position of C71 and the amino group at the 6th position of A29, respectively (Figure 10C). In addition, the activity of F3 + S3 was only exceeded in the case of U30 + A47, demonstrating that this combination further stabilized the active site.

Ligase ribozymes catalyze the nucleophilic attack by a 3′-hydroxyl on a 5′-α-phosphorus of triphosphates to form a 3′-5′-phosphodiester bond with the concomitant release of pyrophosphate. Considering their tertiary structure, ligase ribozymes have a simple three-helix junction architecture at their active sites [19,26]. Despite similar conformations, the catalytic rate of the class I ligase ribozyme is of the order of 100 per minute, comparable to that found in natural proteinaceous polymerases [12,38], which is orders of magnitude faster than that of other RNA enzymes, such as R3C ligase [9], L1 ligase [10], and DSL ligase [11]. In proteinaceous enzymes, it is thought that one Mg^2+^ lowers the p*K*_a_ of the 3′-hydroxyl for an in-line nucleophilic attack and that the other Mg^2+^ assists pyrophosphate release by stabilizing the negative charge [40]. Elucidating the causes of ligase ribozyme activity, how its activity rate increases, and its replacement with a protein ligase will be an important theme in future evolutionary biology.

## 5. Conclusions

The class I ligase ribozyme comprises 121 nucleotides and forms seven paired regions (P1–P7). Although a mutant with a P7 complete removal showed significantly reduced activity, a P7 deletion mutant with a GAAA tetraloop showed almost the same ligation level as that of the original class I ligase. In contrast, the P5 moiety is completely removed. Regions P2, P4, and J3/4 are required for this activity. The class I ligase ribozyme can be divided into two molecules in the P5 region. The ligation activity was retained even when splitting into two molecules of 55 and 39 nucleotides. We also analyzed the ligation activity of mutants with active sites C30, C47, and A71 using split ribozymes (F3 + S3). The C30 substitution had a greater effect on ligation activity in the order of G, A, and U, and the A71 substitution caused a greater effect on ligation activity in the order of G, C, and U. When C47 was replaced with A, the ligation activity was close to that of F3 + S3, and the A47 mutants showed similar activities upon the further replacement of C30 with any other base. Our findings suggest the possibility of structural–functional architecture formation in short RNA molecules’ base on the class I ligase ribozyme’s assembling from multiple fragments that would be achievable with prebiotic synthesis.

## Data Availability

The data supporting the findings of this study are shown in the manuscript.

## References

[1] Crick F.H.C. (1958). On protein synthesis. Symp. Soc. Exp. Biol..

[2] Temin H.M., Mizutani S. (1970). RNA-dependent DNA polymerase in virions of Rous sarcoma virus. Nature.

[3] Kruger K., Grabowski P.J., Zaug A.J., Sands J., Gottschling D.E., Cech T.R. (1982). Self-splicing RNA: Autoexcision and autocyclization of the ribosomal RNA intervening sequence of Tetrahymena. Cell.

[4] Guerrier-Takada C., Gardiner K., Marsh T., Pace N., Altman S. (1983). The RNA moiety of ribonuclease P is the catalytic subunit of the enzyme. Cell.

[5] Gilbert W. (1986). Origin of life: The RNA world. Nature.

[6] Tamura K. (2015). Origins and early evolution of the tRNA molecule. Life.

[7] Zielinski W., Orgel L. (1987). Autocatalytic synthesis of a tetranucleotide analogue. Nature.

[8] Sievers D., von Kiedrowski G. (1994). Self-replication of complementary nucleotide-based oligomers. Nature.

[9] Rogers J., Joyce G.F. (2001). The effect of cytidine on the structure and function of an RNA ligase ribozyme. RNA.

[10] Robertson M.P., Ellington A.D. (1999). In vitro selection of an allosteric ribozyme that transduces analytes to amplicons. Nat. Biotechnol..

[11] Ikawa Y., Tsuda K., Matsumura S., Inoue T. (2004). De novo synthesis and development of an RNA enzyme. Proc. Natl. Acad. Sci. USA.

[12] Ekland E.H., Szostak J.W., Bartel D.P. (1995). Structurally complex and highly active RNA ligases derived from random RNA sequences. Science.

[13] Johnston W.K., Unrau P.J., Lawrence M.S., Glasner M.E., Bartel D.P. (2001). RNA-catalyzed RNA polymerization: Accurate and general RNA-templated primer extension. Science.

[14] Lawrence M.S., Bartel D.P. (2005). New ligase-derived RNA polymerase ribozymes. RNA.

[15] Zaher H.S., Unrau P.J. (2007). Selection of an improved RNA polymerase ribozyme with superior extension and fidelity. RNA.

[16] Ellington A.D., Szostak J.W. (1990). In vitro selection of RNA molecules that bind specific ligands. Nature.

[17] Tuerk C., Gold L. (1990). Systematic evolution of ligands by exponential enrichment: RNA ligands to bacteriophage T4 DNA polymerase. Science.

[18] Robertson M.P., Scott W.G. (2007). The structural basis of ribozyme-catalyzed RNA assembly. Science.

[19] Kurihara E., Uchida S., Umehara T., Tamura K. (2014). Development of a functionally minimized mutant of the R3C ligase ribozyme offers insight into the plausibility of the RNA world hypothesis. Biology.

[20] Nomura Y., Yokobayashi Y. (2019). Systematic minimization of RNA ligase ribozyme through large-scale design-synthesis-sequence cycles. Nucleic Acids Res..

[21] Tanizawa K., Uchida S., Kurihara E., Umehara T., Tamura K. (2018). The kiss switch brings inactive R3C ligase ribozyme back to life. Biology.

[22] Hamachi K., Mutsuro-Aoki H., Tanizawa K., Hirasawa I., Umehara T., Tamura K. (2019). Effects of complementary loop composition in truncated R3C ligase ribozymes on kiss switch activation. Biosystems.

[23] Mutsuro-Aoki H., Hamachi K., Kurihara R., Tamura K. (2020). Aminoacylation of short hairpin RNAs through kissing-loop interactions indicates evolutionary trend of RNA molecules. Biosystems.

[24] Mutsuro-Aoki H., Tamura K. (2022). Acquisition of dual ribozyme-functions in nonfunctional short hairpin RNAs through kissing-loop interactions. Life.

[25] Shechner D.M., Grant R.A., Bagby S.C., Koldobskaya Y., Piccirilli J.A., Bartel D.P. (2009). Crystal structure of the catalytic core of an RNA-polymerase ribozyme. Science.

[26] Shechner D.M., Bartel D.P. (2011). The structural basis of RNA-catalyzed RNA polymerization. Nat. Struct. Mol. Biol..

[27] Eigen M., Schuster P. (1977). Hypercycle. A principle of natural self-organization. Part A: Emergence of the hypercycle. Naturwissenschaften.

[28] Ferris J.P. (2006). Montmorillonite-catalysed formation of RNA oligomers: The possible role of catalysis in the origins of life. Phil. Trans. R. Soc. B.

[29] Robertson M.P., Hesselberth J.R., Ellington A.D. (2001). Optimization and optimality of a short ribozyme ligase that joins non-Watson-Crick base pairings. RNA.

[30] Hayden E.J., Lehman N. (2006). Self-assembly of a group I intron from inactive oligonucleotide fragments. Chem. Biol..

[31] Wachowius F., Holliger P. (2019). Non-enzymatic assembly of a minimized RNA polymerase ribozyme. ChemSystemsChem.

[32] Akoopie A., Müller U.F. (2016). Lower temperature optimum of a smaller, fragmented triphosphorylation ribozyme. Phys. Chem. Chem. Phys..

[33] Bergman N.H., Lau N.C., Lehnert V., Westhof E., Bartel D.P. (2004). The three-dimensional architecture of the class I ligase ribozyme. RNA.

[34] Hamachi K., Hayashi H., Shimamura M., Yamaji Y., Kaneko A., Fujisawa A., Umehara T., Tamura K. (2013). Glycols modulate terminator stem stability and ligand-dependency of a glycine riboswitch. BioSystems.

[35] Jaeger L., Michel F., Westhof E. (1994). Involvement of a GNRA tetraloop in long-range RNA tertiary interaction. J. Mol. Biol..

[36] Rohatgi R., Bartel D.P., Szostak J.W. (1996). Kinetic and mechanistic analysis of nonenzymatic, template-directed oligoribonucleotide ligation. J. Am. Chem. Soc..

[37] Tinoco I., Borer P.N., Dengler B., Levin M.D., Uhlenbeck O.C., Crothers D.M., Bralla J. (1973). Improved estimation of secondary structure in ribonucleic acids. Nat. New Biol..

[38] Joyce G.F. (2007). A glimpse of biology’s first enzyme. Science.

[39] Saenger W. (1984). Principles of Nucleic Acid Structure.

[40] Castro C., Smidansky E.D., Arnold J.J., Maksimchuk K.R., Moustafa I., Uchida A., Götte M., Konigsberg W., Cameron C.E. (2009). Nucleic acid polymerases use a general acid for nucleotidyl transfer. Nat. Struct. Mol. Biol..

